# Supplementary material for: The relationship of low-density lipoprotein cholesterol and all-cause or cardiovascular mortality in patients with type 2 diabetes: a retrospective study
Source: PeerJ. 2023 Jan 9;11:e14609. doi: 10.7717/peerj.14609 (PMC9835695; doi:10.7717/peerj.14609)
Supplement: Supplemental Information 1 — 1 LDL-C: low-density cholesterol; 2 Based on Poisson assumption, CI =confidence interval; 3 HR = hazard ratio; CI =confidence interval4 Based on Cox proportional hazard regression with adjusting for general characteristics (i.e., age, and sex) 5 Based on Cox proportional hazard regression with adjusting for the general characteristics adjusted in Model 1 plus the antidiabetic, antihypertensive, and antilipid medications presented in Table 1.6 Based on Cox proportional hazard regression with all covariates included in Model 2 plus comorbidities, complications, and laboratory results presented in Table 1B values for the interaction of mean LDL-C with statins and fibrates were 0.07. [file peerj-11-14609-s001.docx]

Supplemental Table 1: Overall and mean albumin-specific rates and relative hazard ratios of all-cause mortality by mean low-density lipoprotein cholesterol percentile (<10^th^, 10^th^ - 25^th^, 25^th^ - 50^th^, 50^th^ - 75^th^, 75^th^ - 90^th^, >90^th^) in patients with type 2 diabetes

| Mean LDL-C  (mg/dL)^1^ | All-cause Mortality | | |  | Model 1  Adjusted HR (95% CI) ^3^ |  | Model 2  Adjusted HR (95% CI) ^3^ |  | Model 3  Adjusted HR (95% CI) ^3^ |
| --- | --- | --- | --- | --- | --- | --- | --- | --- | --- |
|  | No. of patients | No. of mortality | Rates (per 1,000 patient-years)  (95% CI) ^2^ |  |  |  |  |  |  |
| **Overall** |  |  |  |  |  |  |  |  |  |
| ≤77 | 4,317 | 1,630 | 61.15 (58.18-64.12) |  | 2.02 (1.90-2.16)^4^ |  | 1.67 (1.56-1.78)^5^ |  | 1.47 (1.35-1.59)^6^ |
| >77-90 | 6,451 | 1,596 | 34.04 (32.37-35.71) |  | 1.20 (1.13-1.28)^4^ |  | 1.15 (1.08-1.22)^5^ |  | 1.13 (1.05-1.22)^6^ |
| >90-103.59 | 10,710 | 2,237 | 27.58 (26.44-28.72) |  | 1.00 (Reference) |  | 1.00 (Reference) |  | 1.00 (Reference) |
| >103.59-119 | 10,842 | 2,364 | 30.37 (29.15-31.60) |  | 1.20 (1.13-1.27)^4^ |  | 1.24 (1.17-1.31)^5^ |  | 1.14 (1.07-1.22)^6^ |
| >119-135.5 | 6,336 | 1,492 | 36.72 (34.85-38.58) |  | 1.55 (1.45-1.66)^4^ |  | 1.66 (1.56-1.78)^5^ |  | 1.33 (1.23-1.44)^6^ |
| >135.59 | 4,295 | 1,322 | 55.81 (52.80-58.82) |  | 2.64 (2.47-2.83)^4^ |  | 2.85 (2.66-3.05)^5^ |  | 1.52 (1.38-1.68)^6^ |
| **Mean Albumin ≥3.5 g/dL** |  |  |  |  |  |  |  |  |  |
| ≤77 | 2,023 | 607 | 41.92 (38.59-45.26) |  | 1.64 (1.49-1.81)^4^ |  | 1.42 (1.28-1.57)^5^ |  | 1.61 (1.43-1.81)^6^ |
| >77-90 | 3,305 | 708 | 26.10 (24.17-28.02) |  | 1.13 (1.03-1.24)^4^ |  | 1.08 (0.98-1.19)^5^ |  | 1.19 (1.08-1.32)^6^ |
| >90-103.59 | 5,686 | 1,119 | 23.12 (21.77-24.48) |  | 1.00 (Reference) |  | 1.00 (Reference) |  | 1.00 (Reference) |
| >103.59-119 | 5,521 | 1,200 | 26.76 (25.25-28.28) |  | 1.25 (1.16-1.36)^4^ |  | 1.29 (1.19-1.41)^5^ |  | 1.08 (0.99-1.18)^6^ |
| >119-135.5 | 2,998 | 706 | 32.72 (30.31-35.14) |  | 1.65 (1.50-1.82)^4^ |  | 1.78 (1.62-1.95)^5^ |  | 1.12 (1.00-1.26)^6^ |
| >135.59 | 1,755 | 515 | 47.38 (43.29-51.47) |  | 2.80 (2.52-3.11)^4^ |  | 2.97 (2.67-3.30)^5^ |  | 1.18 (1.01-1.38)^6^ |
| **Mean Albumin 3.0-3.4 g/dL** |  |  |  |  |  |  |  |  |  |
| ≤77 | 633 | 440 | 115.51 (104.71-126.30) |  | 1.49 (1.31-1.69)^4^ |  | 1.33 (1.17-1.51)^5^ |  | 1.45 (1.24-1.70)^6^ |
| >77-90 | 647 | 413 | 97.05 (87.69-106.41) |  | 1.18 (1.04-1.35)^4^ |  | 1.19 (1.04-1.36)^5^ |  | 1.19 (1.03-1.38)^6^ |
| >90-103.59 | 825 | 487 | 83.24 (75.85-90.63) |  | 1.00 (Reference) |  | 1.00 (Reference) |  | 1.00 (Reference) |
| >103.59-119 | 748 | 439 | 83.78 (75.94-91.62) |  | 1.12 (0.98-1.27)^4^ |  | 1.15 (1.01-1.31)^5^ |  | 1.01 (0.88-1.17)^6^ |
| >119-135.5 | 483 | 301 | 92.16 (81.75-102.57) |  | 1.28 (1.11-1.48)^4^ |  | 1.37 (1.19-1.59)^5^ |  | 1.04 (0.88-1.24)^6^ |
| >135.59 | 451 | 293 | 102.71 (90.95-114.47) |  | 1.62 (1.40-1.88)^4^ |  | 1.77 (1.53-2.05)^5^ |  | 1.20 (0.98-1.46)^6^ |
| **Mean Albumin 2.5-2.9 g/dL** |  |  |  |  |  |  |  |  |  |
| ≤77 | 346 | 287 | 161.52 (142.84-180.21) |  | 1.71 (1.43-2.03)^4^ |  | 1.49 (1.25-1.78)^5^ |  | 1.43 (1.14-1.79)^6^ |
| >77-90 | 269 | 185 | 126.30 (108.10-144.50) |  | 1.17 (0.96-1.42)^4^ |  | 1.13 (0.93-1.38)^5^ |  | 1.03 (0.82-1.29)^6^ |
| >90-103.59 | 367 | 233 | 101.25 (88.25-114.25) |  | 1.00 (Reference) |  | 1.00 (Reference) |  | 1.00 (Reference) |
| >103.59-119 | 348 | 235 | 104.45 (91.09-117.80) |  | 1.06 (0.88-1.27)^4^ |  | 1.09 (0.91-1.31)^5^ |  | 1.02 (0.83-1.26)^6^ |
| >119-135.5 | 212 | 147 | 121.47 (101.83-141.11) |  | 1.33 (1.08-1.64)^4^ |  | 1.38 (1.12-1.70)^5^ |  | 1.18 (0.92-1.52)^6^ |
| >135.59 | 226 | 155 | 123.42 (103.99-142.85) |  | 1.52 (1.23-1.86)^4^ |  | 1.68 (1.36-2.07)^5^ |  | 1.16 (0.85-1.58)^6^ |
| **Mean Albumin <2.5 g/dL** |  |  |  |  |  |  |  |  |  |
| ≤77 | 138 | 119 | 205.27 (168.39-242.15) |  | 2.24 (1.71-2.93)^4^ |  | 1.90 (1.44-2.52)^5^ |  | 2.34 (1.64-3.35)^6^ |
| >77-90 | 116 | 77 | 111.96 (86.95-136.97) |  | 1.16 (0.86-1.57)^4^ |  | 1.09 (0.81-1.48)^5^ |  | 1.20 (0.84-1.71)^6^ |
| >90-103.59 | 153 | 96 | 102.14 (81.71-122.57) |  | 1.00 (Reference) |  | 1.00 (Reference) |  | 1.00 (Reference) |
| >103.59-119 | 148 | 105 | 113.44 (91.74-135.13) |  | 1.08 (0.82-1.43)^4^ |  | 1.12 (0.84-1.47)^5^ |  | 1.36 (0.98-1.88)^6^ |
| >119-135.5 | 110 | 82 | 148.55 (116.40-180.71) |  | 1.61 (1.20-2.16)^4^ |  | 1.62 (1.20-2.18)^5^ |  | 1.84 (1.29-2.63)^6^ |
| >135.59 | 130 | 97 | 147.84 (118.42-177.26) |  | 1.87 (1.40-2.50)^4^ |  | 2.15 (1.60-2.89)^5^ |  | 2.16 (1.45-3.22)^6^ |

^1^ LDL-C: low-density cholesterol

^2^ Based on Poisson assumption, CI=confidence interval

^3^ HR= hazard ratio; CI=confidence interval

^4^ Based on Cox proportional hazard regression adjusting for general characteristics (i.e., , age, and sex)

^5^ Based on Cox proportional hazard regression adjusting for the general characteristics in Model 1 plus the antidiabetic, antihypertensive, and antilipid medications presented in Table 1.

^6^ Based on Cox proportional hazard regression with all covariates included in Model 2 plus comorbidities, complications, and laboratory results presented in Table 1.

*P* value for the interaction of mean LDL-C with albumin was <0.001.
