# Supplementary material for: The relationship of low-density lipoprotein cholesterol and all-cause or cardiovascular mortality in patients with type 2 diabetes: a retrospective study
Source: PeerJ. 2023 Jan 9;11:e14609. doi: 10.7717/peerj.14609 (PMC9835695; doi:10.7717/peerj.14609)
Supplement: Supplemental Information 2 — 1 LDL-C: low-density cholesterol2 Based on Poisson distribution, CI =confidence interval; 3 HR = hazard ratio; CI =confidence interval4 Based on Cox proportional hazard regression adjusting for general characteristics (i.e., diabetes type, age, and sex) 5 Based on Cox proportional hazard regression adjusting for the general characteristics in Model 1 plus the antidiabetic, antihypertensive, and antilipids medications presented in Table 1.6 Based on Cox proportional hazard regression with all covariates included in Model 2 plus comorbidities, complications, and laboratory results presented in Table 1.P value for the interaction of mean LDL-C with mean albumin was 0.0121. [file peerj-11-14609-s002.docx]

Supplemental Table 2: Overall and mean albumin-specific rates and relative hazard ratios of cardiovascular mortality by mean low-density lipoprotein cholesterol percentile (<10^th^, 10^th^ - 25^th^, 25^th^ - 50^th^, 50^th^ - 75^th^, 75^th^ - 90^th^, >90^th^) in patients with type 2 diabetes

| Mean LDL-C  (mg/dL)^1^ | Cardiovascular Mortality | | |  | Model 1  Adjusted HR (95% CI) ^3^ |  | Model 2  Adjusted HR (95% CI) ^3^ |  | Model 3  Adjusted HR (95% CI) ^3^ |
| --- | --- | --- | --- | --- | --- | --- | --- | --- | --- |
|  | No. of patients | No. of mortality | Rates (per 1,000 patient-years)  (95% CI) ^2^ |  |  |  |  |  |  |
| **Overall** |  |  |  |  |  |  |  |  |  |
| ≤77 | 4,317 | 296 | 11.10 (9.84-12.37) |  | 1.85 (1.60-2.15)^4^ |  | 1.61 (1.38-1.87)^5^ |  | 1.48 (1.23-1.78)^6^ |
| >77-90 | 6,451 | 296 | 6.31 (5.59-7.03) |  | 1.12 (0.97-1.30)^4^ |  | 1.09 (0.94-1.26)^5^ |  | 1.13 (0.95-1.34)^6^ |
| >90-103.59 | 10,710 | 444 | 5.47 (4.96-5.98) |  | 1.00 (Reference) |  | 1.00 (Reference) |  | 1.00 (Reference) |
| >103.59-119 | 10,842 | 503 | 6.46 (5.90-7.03) |  | 1.29 (1.13-1.46)^4^ |  | 1.32 (1.16-1.50)^5^ |  | 1.18 (1.02-1.38)^6^ |
| >119-135.5 | 6,336 | 306 | 7.53 (6.69-8.37) |  | 1.61 (1.40-1.87)^4^ |  | 1.70 (1.47-1.97)^5^ |  | 1.34 (1.12-1.61)^6^ |
| >135.59 | 4,295 | 278 | 11.74 (10.36-13.12) |  | 2.83 (2.44-3.29)^4^ |  | 3.02 (2.60-3.51)^5^ |  | 1.51 (1.21-1.89)^6^ |
| **Mean Albumin ≥3.5 g/dL** |  |  |  |  |  |  |  |  |  |
| ≤77 | 2,023 | 124 | 8.56 (7.06-10.07) |  | 1.66 (1.33-2.07)^4^ |  | 1.52 (1.21-1.89)^5^ |  | 1.84 (1.43-2.37)^6^ |
| >77-90 | 3,305 | 163 | 6.01 (5.09-6.93) |  | 1.27 (1.04-1.56)^4^ |  | 1.25 (1.02-1.52)^5^ |  | 1.34 (1.08-1.68)^6^ |
| >90-103.59 | 5,686 | 228 | 4.71 (4.10-5.32) |  | 1.00 (Reference) |  | 1.00 (Reference) |  | 1.00 (Reference) |
| >103.59-119 | 5,521 | 285 | 6.36 (5.62-7.09) |  | 1.46 (1.22-1.73)^4^ |  | 1.48 (1.25-1.77)^5^ |  | 1.24 (1.03-1.51)^6^ |
| >119-135.5 | 2,998 | 148 | 6.86 (5.75-7.97) |  | 1.69 (1.38-2.08)^4^ |  | 1.76 (1.43-2.17)^5^ |  | 1.23 (0.96-1.56)^6^ |
| >135.59 | 1,755 | 117 | 10.76 (8.81-12.72) |  | 3.10 (2.48-3.88)^4^ |  | 3.20 (2.55-4.00)^5^ |  | 1.28 (0.92-1.78)^6^ |
| **Mean Albumin 3.0-3.4 g/dL** |  |  |  |  |  |  |  |  |  |
| ≤77 | 633 | 84 | 22.05 (17.34-26.77) |  | 1.29 (0.97-1.72)^4^ |  | 1.18 (0.88-1.58)^5^ |  | 1.29 (0.90-1.85)^6^ |
| >77-90 | 647 | 63 | 14.80 (11.15-18.46) |  | 0.82 (0.60-1.12)^4^ |  | 0.83 (0.61-1.13)^5^ |  | 0.92 (0.66-1.30)^6^ |
| >90-103.59 | 825 | 107 | 18.29 (14.82-21.75) |  | 1.00 (Reference) |  | 1.00 (Reference) |  | 1.00 (Reference) |
| >103.59-119 | 748 | 76 | 14.50 (11.24-17.77) |  | 0.88 (0.65-1.18)^4^ |  | 0.89 (0.67-1.20)^5^ |  | 0.70 (0.50-0.98)^6^ |
| >119-135.5 | 483 | 54 | 16.53 (12.12-20.94) |  | 1.05 (0.76-1.46)^4^ |  | 1.15 (0.83-1.60)^5^ |  | 0.78 (0.53-1.16)^6^ |
| >135.59 | 451 | 63 | 22.08 (16.63-27.54) |  | 1.60 (1.17-2.19)^4^ |  | 1.69 (1.23-2.33)^5^ |  | 0.97 (0.62-1.52)^6^ |
| **Mean Albumin 2.5-2.9 g/dL** |  |  |  |  |  |  |  |  |  |
| ≤77 | 346 | 44 | 24.76 (17.45-32.08) |  | 1.66 (1.07-2.59)^4^ |  | 1.61 (1.02-2.53)^5^ |  | 1.23 (0.71-2.14)^6^ |
| >77-90 | 269 | 36 | 24.58 (16.55-32.61) |  | 1.46 (0.92-2.32)^4^ |  | 1.46 (0.91-2.32)^5^ |  | 1.15 (0.67-1.95)^6^ |
| >90-103.59 | 367 | 36 | 15.64 (10.53-20.75) |  | 1.00 (Reference) |  | 1.00 (Reference) |  | 1.00 (Reference) |
| >103.59-119 | 348 | 50 | 22.22 (16.06-28.38) |  | 1.47 (0.96-2.25)^4^ |  | 1.47 (0.96-2.26)^5^ |  | 1.16 (0.72-1.88)^6^ |
| >119-135.5 | 212 | 36 | 29.75 (20.03-39.47) |  | 2.14 (1.34-3.40)^4^ |  | 2.17 (1.36-3.45)^5^ |  | 1.49 (0.86-2.60)^6^ |
| >135.59 | 226 | 33 | 26.28 (17.31-35.24) |  | 2.14 (1.33-3.45)^4^ |  | 2.10 (1.30-3.40)^5^ |  | 1.18 (0.59-2.37)^6^ |
| **Mean Albumin <2.5 g/dL** |  |  |  |  |  |  |  |  |  |
| ≤77 | 138 | 14 | 24.15 (11.50-36.80) |  | 1.52 (0.74-3.13)^4^ |  | 1.35 (0.64-2.85)^5^ |  | 1.80 (0.67-4.88)^6^ |
| >77-90 | 116 | 8 | 11.63 (3.57-19.69) |  | 0.78 (0.33-1.83)^4^ |  | 0.72 (0.31-1.70)^5^ |  | 0.63 (0.19-2.03)^6^ |
| >90-103.59 | 153 | 16 | 17.02 (8.68-23.37) |  | 1.00 (Reference) |  | 1.00 (Reference) |  | 1.00 (Reference) |
| >103.59-119 | 148 | 22 | 23.77 (13.84-33.70) |  | 1.39 (0.73-2.66)^4^ |  | 1.47 (0.77-2.83)^5^ |  | 1.96 (0.88-4.36)^6^ |
| >119-135.5 | 110 | 16 | 28.99 (14.78-43.19) |  | 1.95 (0.97-3.93)^4^ |  | 2.02 (1.00-4.08)^5^ |  | 2.72 (1.13-6.56)^6^ |
| >135.59 | 130 | 12 | 18.29 (7.94-28.64) |  | 1.58 (0.73-3.40)^4^ |  | 1.76 (0.81-3.84)^5^ |  | 1.73 (0.60-5.02)^6^ |

^1^ LDL-C: low-density cholesterol

^2^ Based on Poisson assumption, CI=confidence interval

^3^ HR= hazard ratio; CI=confidence interval

^4^ Based on Cox proportional hazard regression adjusting for general characteristics (i.e., , age, and sex)

^5^ Based on Cox proportional hazard regression adjusting for the general characteristics in Model 1 plus the antidiabetic, antihypertensive, and antilipids medications presented in Table 1.

^6^ Based on Cox proportional hazard regression with all covariates included in Model 2 plus comorbidities, complications, and laboratory results presented in Table 1.

*P* value for the interaction of mean LDL-C with mean albumin was 0.0121.
