# Supplementary material for: The relationship of low-density lipoprotein cholesterol and all-cause or cardiovascular mortality in patients with type 2 diabetes: a retrospective study
Source: PeerJ. 2023 Jan 9;11:e14609. doi: 10.7717/peerj.14609 (PMC9835695; doi:10.7717/peerj.14609)
Supplement: Supplemental Information 3 — 1 LDL-C: low-density cholesterol2 Based on Poisson distribution, CI =confidence interval; 3 HR = hazard ratio; CI =confidence interval4 Based on Cox proportional hazard regression adjusting for general characteristics (i.e., diabetes type, age, and sex) 5 Based on Cox proportional hazard regression adjusting for the general characteristics in Model 1 plus the antidiabetic, antihypertensive, and antilipid medications presented in Table 1.6 Based on Cox proportional hazard regression with all covariates included in Model 2 plus comorbidities, complications, and laboratory results presented in Table 1.P values for the interaction of mean LDL-C with sex for all-cause and cardiovascular mortality were < 0.0001 and 0.0003, respectively. [file peerj-11-14609-s003.docx]

Supplemental Table 3: Sex-specific rates and relative hazard ratios of all-cause and cardiovascular mortality by mean low-density lipoprotein cholesterol percentile (<10^th^, 10^th^ - 25^th^, 25^th^ - 50^th^, 50^th^ - 75^th^, 75^th^ - 90^th^, >90^th^) in patients with type 2 diabetes

| Mean LDL-C  (mg/dL)^1^ | Mortality | | |  | Model 1  Adjusted HR (95% CI) ^3^ |  | Model 2  Adjusted HR (95% CI) ^3^ |  | Model 3  Adjusted HR (95% CI) ^3^ |
| --- | --- | --- | --- | --- | --- | --- | --- | --- | --- |
|  | No. of patients | No. of mortality | Rates (per 1,000 patient-years)  (95% CI) ^2^ |  |  |  |  |  |  |
| **All-cause Mortality**  **Men** |  |  |  |  |  |  |  |  |  |
| ≤77 | 2,565 | 937 | 60.32 (56.45-64.18) |  | 2.04 (1.88-2.23)^4^ |  | 1.69 (1.55-1.84)^5^ |  | 1.55 (1.38-1.73)^6^ |
| >77-90 | 3,595 | 835 | 32.23 (30.05-34.42) |  | 1.11 (1.01-1.21)^4^ |  | 1.05 (0.96-1.15)^5^ |  | 1.11 (1.00-1.23)^6^ |
| >90-103.59 | 5,735 | 1,219 | 28.89 (27.27-30.51) |  | 1.00 (Reference) |  | 1.00 (Reference) |  | 1.00 (Reference) |
| >103.59-119 | 5,705 | 1,243 | 31.24 (29.50-32.97) |  | 1.18 (1.09-1.28)^4^ |  | 1.22 (1.12-1.32)^5^ |  | 1.07 (0.97-1.18)^6^ |
| >119-135.5 | 3,254 | 765 | 37.84 (35.15-40.52) |  | 1.56 (1.43-1.71)^4^ |  | 1.65 (1.51-1.81)^5^ |  | 1.21 (1.08-1.37)^6^ |
| >135.5 | 2,170 | 652 | 56.15 (51.84-60.46) |  | 2.58 (2.35-2.84)^4^ |  | 2.77 (2.52-3.05)^5^ |  | 1.25 (1.07-1.45)^6^ |
| **Women** |  |  |  |  |  |  |  |  |  |
| ≤77 | 1,744 | 693 | 62.56 (57.90-67.22) |  | 1.99 (1.81-2.20)^4^ |  | 1.64 (1.48-1.80)^5^ |  | 1.50 (1.33-1.69)^6^ |
| >77-90 | 2,841 | 761 | 36.47 (33.88-39.07) |  | 1.33 (1.21-1.46)^4^ |  | 1.27 (1.15-1.39)^5^ |  | 1.20 (1.07-1.34)^6^ |
| >90-103.59 | 4,968 | 1,018 | 26.19 (24.58-27.80) |  | 1.00 (Reference) |  | 1.00 (Reference) |  | 1.00 (Reference) |
| >103.59-119 | 5,124 | 1,121 | 29.56 (27.83-31.29) |  | 1.21 (1.12-1.32)^4^ |  | 1.27 (1.16-1.38)^5^ |  | 1.19 (1.08-1.32)^6^ |
| >119-135.5 | 3,074 | 727 | 35.70 (33.11-38.30) |  | 1.54 (1.40-1.69)^4^ |  | 1.68 (1.53-1.85)^5^ |  | 1.38 (1.23-1.55)^6^ |
| >135.5 | 2,119 | 670 | 55.70 (51.48-59.92) |  | 2.71 (2.46-2.99)^4^ |  | 2.94 (2.66-3.24)^5^ |  | 1.72 (1.49-1.98)^6^ |
| **Cardiovascular Mortality**  **Men** |  |  |  |  |  |  |  |  |  |
| ≤77 | 2,565 | 161 | 10.36 (8.76-11.97) |  | 1.79 (1.46-2.18)^4^ |  | 1.57 (1.28-1.92)^5^ |  | 1.54 (1.19-1.99)^6^ |
| >77-90 | 3,595 | 158 | 6.10 (5.15-7.05) |  | 1.05 (0.86-1.28)^4^ |  | 1.02 (0.84-1.25)^5^ |  | 1.15 (0.91-1.46)^6^ |
| >90-103.59 | 5,735 | 243 | 5.76 (5.04-6.48) |  | 1.00 (Reference) |  | 1.00 (Reference) |  | 1.00 (Reference) |
| >103.59-119 | 5,705 | 267 | 6.71 (5.91-7.51) |  | 1.27 (1.07-1.52)^4^ |  | 1.30 (1.09-1.55)^5^ |  | 1.10 (0.90-1.35)^6^ |
| >119-135.5 | 3,254 | 156 | 7.72 (6.51-8.93) |  | 1.61 (1.31-1.97)^4^ |  | 1.68 (1.38-2.06)^5^ |  | 1.19 (0.91-1.54)^6^ |
| >135.5 | 2,170 | 148 | 12.75 (10.69-14.80) |  | 2.99 (2.43-3.67)^4^ |  | 3.16 (2.57-3.88)^5^ |  | 1.33 (0.96-1.84)^6^ |
| **Women** |  |  |  |  |  |  |  |  |  |
| ≤77 | 1,744 | 135 | 12.19 (10.13-14.24) |  | 1.92 (1.54-2.39)^4^ |  | 1.65 (1.32-2.05)^5^ |  | 1.61 (1.23-2.11)^6^ |
| >77-90 | 2,841 | 138 | 6.61 (5.51-7.72) |  | 1.21 (0.98-1.51)^4^ |  | 1.17 (0.94-1.46)^5^ |  | 1.16 (0.90-1.49)^6^ |
| >90-103.59 | 4,968 | 201 | 5.17 (4.46-5.89) |  | 1.00 (Reference) |  | 1.00 (Reference) |  | 1.00 (Reference) |
| >103.59-119 | 5,124 | 236 | 6.22 (5.43-7.02) |  | 1.30 (1.08-1.57)^4^ |  | 1.35 (1.12-1.63)^5^ |  | 1.21 (0.97-1.51)^6^ |
| >119-135.5 | 3,074 | 150 | 7.37 (6.19-8.55) |  | 1.61 (1.30-1.99)^4^ |  | 1.73 (1.40-2.14)^5^ |  | 1.40 (1.08-1.80)^6^ |
| >135.5 | 2,119 | 130 | 10.81 (8.95-12.67) |  | 2.68 (2.15-3.34)^4^ |  | 2.89 (2.32-3.61)^5^ |  | 1.46 (1.05-2.03)^6^ |

^1^ LDL-C: low-density cholesterol

^2^ Based on Poisson assumption, CI=confidence interval

^3^ HR= hazard ratio; CI=confidence interval

^4^ Based on Cox proportional hazard regression adjusting for general characteristics (i.e., age, and sex)

^5^ Based on Cox proportional hazard regression adjusting for the general characteristics in Model 1 plus the antidiabetic, antihypertensive, and antilipid medications presented in Table 1.

^6^ Based on Cox proportional hazard regression with all covariates included in Model 2 plus comorbidities, complications, and laboratory results presented in Table 1.

*P* values for the interaction of mean LDL-C with sex for all-cause and cardiovascular mortality were < 0.0001 and 0.0003, respectively.
