# Supplementary material for: The relationship of low-density lipoprotein cholesterol and all-cause or cardiovascular mortality in patients with type 2 diabetes: a retrospective study
Source: PeerJ. 2023 Jan 9;11:e14609. doi: 10.7717/peerj.14609 (PMC9835695; doi:10.7717/peerj.14609)
Supplement: Supplemental Information 4 — 1 LDL-C: low-density cholesterol2 Based on Poisson distribution, CI =confidence interval; 3 HR = hazard ratio; CI =confidence interval4 Based on Cox proportional hazard regression adjusting for general characteristics (i.e., diabetes type, age, and sex) 5 Based on Cox proportional hazard regression adjusting for the general characteristics in Model 1 plus the antidiabetic, antihypertensive, and antilipid medications presented in Table 1.6 Based on Cox proportional hazard regression with all covariates included in Model 2 plus comorbidities, complications, and laboratory results presented in Table 1. P values for the interaction of mean LDL-C with age for all-cause and cardiovascular mortality were 0.0367 and 0.8022, respectively. [file peerj-11-14609-s004.docx]

Supplemental Table 4: Age-specific rates and relative hazard ratios of all-cause and cardiovascular mortality by mean low-density lipoprotein cholesterol percentile (<10^th^, 10^th^ - 25^th^, 25^th^ - 50^th^, 50^th^ - 75^th^, 75^th^ - 90^th^, >90^th^) in patients with type 2 diabetes

| Mean LDL-C  (mg/dL)^1^ | Mortality | | |  | Model 1  Adjusted HR (95% CI) ^3^ |  | Model 2  Adjusted HR (95% CI) ^3^ |  | Model 3  Adjusted HR (95% CI) ^3^ |
| --- | --- | --- | --- | --- | --- | --- | --- | --- | --- |
|  | No. of patients | No. of mortality | Rates (per 1,000 patient-years)  (95% CI) ^2^ |  |  |  |  |  |  |
| **All-cause Mortality**  **<50 years** |  |  |  |  |  |  |  |  |  |
| ≤77 | 905 | 190 | 32.05 (27.50-36.61) |  | 3.50 (2.86-4.28)^4^ |  | 2.02 (1.63-2.51)^5^ |  | 1.62 (1.24-2.11)^6^ |
| >77-90 | 1,397 | 128 | 11.73 (9.70-13.76) |  | 1.23 (0.99-1.54)^4^ |  | 1.02 (0.81-1.27)^5^ |  | 1.05 (0.80-1.38)^6^ |
| >90-103.59 | 2,484 | 190 | 9.45 (8.10-10.79) |  | 1.00 (Reference) |  | 1.00 (Reference) |  | 1.00 (Reference) |
| >103.59-119 | 2,821 | 214 | 10.01 (8.67-11.35) |  | 1.08 (0.89-1.32)^4^ |  | 1.12 (0.92-1.36)^5^ |  | 1.08 (0.85-1.36)^6^ |
| >119-135.5 | 1,802 | 133 | 10.81 (8.97-12.64) |  | 1.19 (0.95-1.49)^4^ |  | 1.21 (0.97-1.51)^5^ |  | 0.90 (0.68-1.19)^6^ |
| >135.59 | 1,293 | 184 | 24.86 (21.26-28.45) |  | 3.03 (2.48-3.72)^4^ |  | 3.13 (2.55-3.84)^5^ |  | 0.74 (0.54-1.02)^6^ |
| **50-69 years** |  |  |  |  |  |  |  |  |  |
| ≤77 | 2,113 | 660 | 47.79 (44.14-51.43) |  | 2.35 (2.14-2.60)^4^ |  | 1.90 (1.72-2.09)^5^ |  | 1.42 (1.26-1.60)^6^ |
| >77-90 | 3,568 | 700 | 25.81 (23.90-27.72) |  | 1.17 (1.06-1.29)^4^ |  | 1.13 (1.02-1.24)^5^ |  | 1.05 (0.94-1.18)^6^ |
| >90-103.59 | 6,129 | 1,033 | 21.69 (20.37-23.02) |  | 1.00 (Reference) |  | 1.00 (Reference) |  | 1.00 (Reference) |
| >103.59-119 | 6,172 | 1,173 | 26.04 (24.55-27.53) |  | 1.25 (1.15-1.36)^4^ |  | 1.30 (1.19-1.41)^5^ |  | 1.22 (1.10-1.34)^6^ |
| >119-135.5 | 3,498 | 755 | 33.76 (31.35-36.16) |  | 1.73 (1.57-1.90)^4^ |  | 1.86 (1.70-2.05)^5^ |  | 1.48 (1.32-1.66)^6^ |
| >135.59 | 2,374 | 733 | 55.81 (51.77-59.85) |  | 3.15 (2.86-3.46)^4^ |  | 3.40 (3.09-3.74)^5^ |  | 1.91 (1.66-2.19)^6^ |
| **>69 years** |  |  |  |  |  |  |  |  |  |
| ≤77 | 1,291 | 780 | 113.49 (105.52-121.45) |  | 1.60 (1.46-1.76)^4^ |  | 1.42 (1.29-1.56)^5^ |  | 1.55 (1.37-1.76)^6^ |
| >77-90 | 1,470 | 767 | 87.80 (81.58-94.01) |  | 1.20 (1.10-1.32)^4^ |  | 1.17 (1.06-1.28)^5^ |  | 1.27 (1.13-1.42)^6^ |
| >90-103.59 | 2,091 | 1,014 | 75.96 (71.29-80.64) |  | 1.00 (Reference) |  | 1.00 (Reference) |  | 1.00 (Reference) |
| >103.59-119 | 1,834 | 975 | 86.52 (81.09-91.95) |  | 1.17 (1.07-1.28)^4^ |  | 1.19 (1.09-1.30)^5^ |  | 1.05 (0.94-1.17)^6^ |
| >119-135.5 | 1,028 | 604 | 102.27 (94.12-110.43) |  | 1.44 (1.31-1.60)^4^ |  | 1.53 (1.38-1.69)^5^ |  | 1.21 (1.06-1.39)^6^ |
| >135.59 | 622 | 405 | 130.51 (117.79-143.22) |  | 1.98 (1.76-2.22)^4^ |  | 2.11 (1.88-2.37)^5^ |  | 1.31 (1.09-1.57)^6^ |
| **Cardiovascular Mortality**  **<50 years** |  |  |  |  |  |  |  |  |  |
| ≤77 | 905 | 17 | 2.87 (1.51-4.23) |  | 1.31 (0.75-2.29)^4^ |  | 1.07 (0.60-1.93)^5^ |  | 0.83 (0.41-1.66)^6^ |
| >77-90 | 1,397 | 15 | 1.38 (0.68-2.07) |  | 0.61 (0.34-1.09)^4^ |  | 0.59 (0.33-1.07)^5^ |  | 0.69 (0.35-1.34)^6^ |
| >90-103.59 | 2,484 | 45 | 2.24 (1.58-2.89) |  | 1.00 (Reference) |  | 1.00 (Reference) |  | 1.00 (Reference) |
| >103.59-119 | 2,821 | 48 | 2.25 (1.61-2.88) |  | 1.04 (0.69-1.56)^4^ |  | 1.07 (0.71-1.61)^5^ |  | 0.83 (0.51-1.33)^6^ |
| >119-135.5 | 1,802 | 29 | 2.36 (1.50-3.21) |  | 1.08 (0.67-1.72)^4^ |  | 1.10 (0.69-1.76)^5^ |  | 0.67 (0.38-1.18)^6^ |
| >135.59 | 1,293 | 41 | 5.54 (3.84-7.23) |  | 3.06 (2.00-4.67)^4^ |  | 3.25 (2.12-4.98)^5^ |  | 0.58 (0.30-1.11)^6^ |
| **50-69 years** |  |  |  |  |  |  |  |  |  |
| ≤77 | 2,113 | 120 | 8.69 (7.13-10.24) |  | 2.15 (1.72-2.70)^4^ |  | 1.83 (1.45-2.30)^5^ |  | 1.41 (1.06-1.86)^6^ |
| >77-90 | 3,568 | 132 | 4.87 (4.04-5.70) |  | 1.11 (0.89-1.38)^4^ |  | 1.08 (0.87-1.34)^5^ |  | 1.09 (0.85-1.40)^6^ |
| >90-103.59 | 6,129 | 206 | 4.33 (3.74-4.92) |  | 1.00 (Reference) |  | 1.00 (Reference) |  | 1.00 (Reference) |
| >103.59-119 | 6,172 | 247 | 5.48 (4.80-6.17) |  | 1.32 (1.10-1.59)^4^ |  | 1.37 (1.14-1.65)^5^ |  | 1.31 (1.06-1.62)^6^ |
| >119-135.5 | 3,498 | 149 | 6.66 (5.59-7.73) |  | 1.73 (1.40-2.14)^4^ |  | 1.84 (1.49-2.28)^5^ |  | 1.42 (1.10-1.84)^6^ |
| >135.59 | 2,374 | 157 | 11.95 (10.08-13.82) |  | 3.45 (2.80-4.24)^4^ |  | 3.68 (2.98-4.53)^5^ |  | 1.81 (1.34-2.45)^6^ |
| **>69 years** |  |  |  |  |  |  |  |  |  |
| ≤77 | 1,291 | 159 | 23.13 (19.54-26.73) |  | 1.71 (1.39-2.12)^4^ |  | 1.54 (1.24-1.90)^5^ |  | 1.69 (1.28-2.25)^6^ |
| >77-90 | 1,470 | 149 | 17.06 (14.32-19.79) |  | 1.23 (0.99-1.52)^4^ |  | 1.20 (0.97-1.49)^5^ |  | 1.28 (0.99-1.66)^6^ |
| >90-103.59 | 2,091 | 193 | 14.46 (12.42-16.50) |  | 1.00 (Reference) |  | 1.00 (Reference) |  | 1.00 (Reference) |
| >103.59-119 | 1,834 | 207 | 18.37 (15.87-20.87) |  | 1.30 (1.07-1.58)^4^ |  | 1.31 (1.08-1.60)^5^ |  | 1.06 (0.83-1.35)^6^ |
| >119-135.5 | 1,028 | 128 | 21.67 (17.92-25.43) |  | 1.59 (1.28-1.99)^4^ |  | 1.66 (1.32-2.07)^5^ |  | 1.33 (0.99-1.79)^6^ |
| >135.59 | 622 | 80 | 25.78 (20.13-31.43) |  | 2.01 (1.55-2.62)^4^ |  | 2.12 (1.63-2.75)^5^ |  | 1.37 (0.91-2.07)^6^ |

^1^ LDL-C: low-density cholesterol

^2^ Based on Poisson assumption, CI=confidence interval

^3^ HR= hazard ratio; CI=confidence interval

^4^ Based on Cox proportional hazard regression adjusting for general characteristics (i.e., age, and sex)

^5^ Based on Cox proportional hazard regression adjusting for the general characteristics in Model 1 plus the antidiabetic, antihypertensive, and antilipid medications presented in Table 1.

^6^ Based on Cox proportional hazard regression with all covariates included in Model 2 plus comorbidities, complications, and laboratory results presented in Table 1.

*P* values for the interaction of mean LDL-C with age for all-cause and cardiovascular mortality were 0.0367 and 0.8022, respectively.
