# Supplementary material for: The relationship of low-density lipoprotein cholesterol and all-cause or cardiovascular mortality in patients with type 2 diabetes: a retrospective study
Source: PeerJ. 2023 Jan 9;11:e14609. doi: 10.7717/peerj.14609 (PMC9835695; doi:10.7717/peerj.14609)
Supplement: Supplemental Information 5 — 1 LDL-C: low-density cholesterol; 2 Based on Poisson distribution, CI = confidence interval; 3 HR = hazard ratio; CI = confidence interval4 Based on Cox proportional hazard regression adjusting for general characteristics (i.e., diabetes type, age, and sex) 5 Based on Cox proportional hazard regression adjusting for the general characteristics in Model 1 plus the antidiabetic, antihypertensive, and antilipid medications presented in Table 1.6 Based on Cox proportional hazard regression with all covariates included in Model 2 plus comorbidities, complications, and laboratory results presented in Table 1. [file peerj-11-14609-s005.docx]

Supplemental Table 5: Anti-lipid-specific rates and relative hazard ratios of all-cause and cardiovascular mortality by mean low-density lipoprotein cholesterol percentile (<10^th^, 10^th^ - 25^th^, 25^th^ - 50^th^, 50^th^ - 75^th^, 75^th^ - 90^th^, >90^th^) in patients with type 2 diabetes

| Mean LDL-C  (mg/dL)^1^ | Mortality | | |  | Model 1  Adjusted HR (95% CI) ^3^ |  | Model 2  Adjusted HR (95% CI) ^3^ |  | Model 3  Adjusted HR (95% CI) ^3^ |
| --- | --- | --- | --- | --- | --- | --- | --- | --- | --- |
|  | No. of patients | No. of mortality | Rates (per 1,000 patient-years)  (95% CI) ^2^ |  |  |  |  |  |  |
| **All-cause Mortality**  **Statin use only** |  |  |  |  |  |  |  |  |  |
| ≤77 | 1,591 | 398 | 44.25 (39.91-48.60) |  | 1.97 (1.74-2.22)^4^ |  | 2.01 (1.78-2.27)^5^ |  | 1.76 (1.52-2.03)^6^ |
| >77-90 | 3,071 | 527 | 24.63 (22.53-26.73) |  | 1.16 (1.04-1.30)^4^ |  | 1.17 (1.05-1.30)^5^ |  | 1.17 (1.03-1.32)^6^ |
| >90-103.59 | 5,589 | 829 | 20.20 (18.83-21.58) |  | 1.00 (Reference) |  | 1.00 (Reference) |  | 1.00 (Reference) |
| >103.59-119 | 5,639 | 944 | 24.18 (22.63-25.72) |  | 1.29 (1.18-1.42)^4^ |  | 1.32 (1.21-1.45)^5^ |  | 1.09 (0.98-1.21)^6^ |
| >119-135.5 | 3,264 | 680 | 34.17 (31.60-36.74) |  | 1.84 (1.67-2.04)^4^ |  | 1.90 (1.72-2.11)^5^ |  | 1.21 (1.07-1.38)^6^ |
| >135.59 | 2,473 | 694 | 55.82 (51.66-59.97) |  | 3.61 (3.26-4.00)^4^ |  | 3.66 (3.31-4.05)^5^ |  | 1.37 (1.17-1.60)^6^ |
| **Fribrates use only** |  |  |  |  |  |  |  |  |  |
| ≤77 | 384 | 159 | 58.75 (49.62-67.88) |  | 1.38 (1.11-1.72)^4^ |  | 1.31 (1.05-1.64)^5^ |  | 1.41 (1.04-1.90)^6^ |
| >77-90 | 394 | 115 | 39.77 (32.50-47.04) |  | 1.02 (0.80-1.30)^4^ |  | 0.99 (0.78-1.26)^5^ |  | 1.10 (0.82-1.48)^6^ |
| >90-103.59 | 480 | 164 | 46.18 (39.11-53.25) |  | 1.00 (Reference) |  | 1.00 (Reference) |  | 1.00 (Reference) |
| >103.59-119 | 446 | 142 | 46.76 (39.06-54.45) |  | 1.19 (0.95-1.49)^4^ |  | 1.19 (0.95-1.50)^5^ |  | 1.17 (0.88-1.55)^6^ |
| >119-135.5 | 228 | 77 | 64.09 (49.77-78.40) |  | 2.09 (1.59-2.75)^4^ |  | 2.26 (1.72-2.98)^5^ |  | 2.24 (1.56-3.20)^6^ |
| >135.59 | 127 | 59 | 89.85 (66.93-112.78) |  | 2.95 (2.18-3.99)^4^ |  | 2.91 (2.15-3.94)^5^ |  | 1.36 (0.87-2.13)^6^ |
| **Cardiovascular Mortality**  **Statin use only** |  |  |  |  |  |  |  |  |  |
| ≤77 | 1,591 | 104 | 11.56 (9.34-13.79) |  | 2.60 (2.03-3.32)^4^ |  | 2.61 (2.04-3.34)^5^ |  | 2.17 (1.62-2.92)^6^ |
| >77-90 | 3,071 | 116 | 5.42 (4.44-6.41) |  | 1.30 (1.02-1.65)^4^ |  | 1.29 (1.02-1.64)^5^ |  | 1.31 (1.00-1.72)^6^ |
| >90-103.59 | 5,589 | 164 | 4.00 (3.39-4.61) |  | 1.00 (Reference) |  | 1.00 (Reference) |  | 1.00 (Reference) |
| >103.59-119 | 5,639 | 200 | 5.12 (4.41-5.83) |  | 1.38 (1.12-1.70)^4^ |  | 1.41 (1.15-1.74)^5^ |  | 1.23 (0.97-1.56)^6^ |
| >119-135.5 | 3,264 | 158 | 7.94 (6.70-9.18) |  | 2.18 (1.75-2.71)^4^ |  | 2.25 (1.81-2.80)^5^ |  | 1.60 (1.22-2.10)^6^ |
| >135.59 | 2,473 | 152 | 12.23 (10.28-14.17) |  | 3.99 (3.20-4.98)^4^ |  | 4.10 (3.28-5.12)^5^ |  | 1.69 (1.21-2.37)^6^ |
| **Fibrates use only** |  |  |  |  |  |  |  |  |  |
| ≤77 | 384 | 24 | 8.87 (5.32-12.42) |  | 1.13 (0.67-1.93)^4^ |  | 1.11 (0.65-1.91)^5^ |  | 1.19 (0.55-2.60)^6^ |
| >77-90 | 394 | 17 | 5.88 (3.09-8.67) |  | 0.82 (0.46-1.49)^4^ |  | 0.81 (0.45-1.47)^5^ |  | 1.34 (0.68-2.66)^6^ |
| >90-103.59 | 480 | 32 | 9.01 (5.89-12.13) |  | 1.00 (Reference) |  | 1.00 (Reference) |  | 1.00 (Reference) |
| >103.59-119 | 446 | 27 | 8.89 (5.54-12.24) |  | 1.20 (0.71-2.02)^4^ |  | 1.25 (0.74-2.10)^5^ |  | 1.30 (0.66-2.58)^6^ |
| >119-135.5 | 228 | 18 | 14.98 (8.06-21.90) |  | 2.75 (1.52-4.96)^4^ |  | 2.93 (1.62-5.32)^5^ |  | 4.25 (1.95-9.29)^6^ |
| >135.59 | 127 | 10 | 15.23 (5.79-24.67) |  | 2.89 (1.41-5.93)^4^ |  | 2.95 (1.43-6.09)^5^ |  | 1.67 (0.48-5.82)^6^ |

^1^ LDL-C: low-density cholesterol

^2^ Based on Poisson assumption, CI=confidence interval

^3^ HR= hazard ratio; CI=confidence interval

^4^ Based on Cox proportional hazard regression adjusting for general characteristics (i.e., age, and sex)

^5^ Based on Cox proportional hazard regression adjusting for the general characteristics in Model 1 plus the antidiabetic, antihypertensive, and antilipid medications presented in Table 1.

^6^ Based on Cox proportional hazard regression with all covariates included in Model 2 plus comorbidities, complications, and laboratory results presented in Table 1.

*P* values for the interaction of mean LDL-C with statins and fibrates were 0.02655 and 0.7129, respectively.
