# Supplementary material for: The relationship of low-density lipoprotein cholesterol and all-cause or cardiovascular mortality in patients with type 2 diabetes: a retrospective study
Source: PeerJ. 2023 Jan 9;11:e14609. doi: 10.7717/peerj.14609 (PMC9835695; doi:10.7717/peerj.14609)
Supplement: Supplemental Information 6 — 1 LDL-C-SD: standard deviation of low-density cholesterol2 Based on Poisson distribution, CI =confidence interval; 3 HR = hazard ratio; CI =confidence interval4 Based on Cox proportional hazard regression adjusting for general characteristics (i.e., diabetes type, age, and sex) 5 Based on Cox proportional hazard regression adjusting for the general characteristics in Model 1 plus the antidiabetic, antihypertensive, and antilipid medications presented in Table 1.6 Based on Cox proportional hazard regression with all covariates included in Model 2 plus comorbidities, complications, and laboratory results presented in Table 1. P values for the interaction of mean LDL-C-SD with statins and fibrates were 0.0708 and 0.8841, respectively. [file peerj-11-14609-s006.docx]

Supplemental Table 6: Overall and anti-lipid-specific rates and relative hazard ratios of all-cause and cardiovascular mortality by standard deviation of low-density lipoprotein cholesterol (LDL-C-SD) percentile (<10^th^, 10^th^ - 25^th^, 25^th^ - 50^th^, 50^th^ - 75^th^, 75^th^ - 90^th^, >90^th^) in patients with type 2 diabetes

| LDL-C-SD  (mg/dL)^1^ | Mortality | | |  | Model 1  Adjusted HR (95% CI) ^3^ |  | Model 2  Adjusted HR (95% CI) ^3^ |  | Model 3  Adjusted HR (95% CI) ^3^ |
| --- | --- | --- | --- | --- | --- | --- | --- | --- | --- |
|  | No. of patients | No. of mortality | Rates (per 1,000 patient-years)  (95% CI) ^2^ |  |  |  |  |  |  |
| **All-cause Mortality** |  |  |  |  |  |  |  |  |  |
| ≤5.66 | 3,113 | 703 | 35.65 (33.01-38.28) |  | 1.56 (1.43-1.72)^4^ |  | 1.25 (1.13-1.37)^5^ |  | 1.28 (1.15-1.42)^6^ |
| >5.66-9.72 | 5,127 | 849 | 22.11 (20.62-23.60) |  | 1.02 (0.93-1.11)^4^ |  | 0.89 (0.81-0.97)^5^ |  | 0.98 (0.89-1.08)^6^ |
| >9.72-15.76 | 8,241 | 1,358 | 20.67 (19.57-21.77) |  | 1.00 (Reference) |  | 1.00 (Reference) |  | 1.00 (Reference) |
| >15.76-24.21 | 8,240 | 1,418 | 21.66 (20.53-22.78) |  | 1.07 (1.00-1.16)^4^ |  | 1.17 (1.08-1.26)^5^ |  | 1.01 (0.93-1.09)^6^ |
| >24.21-35.36 | 4,935 | 1,006 | 28.67 (26.90-30.44) |  | 1.50 (1.38-1.63)^4^ |  | 1.68 (1.55-1.82)^5^ |  | 1.23 (1.12-1.35)^6^ |
| >35.36 | 3,305 | 932 | 51.08 (47.81-54.37) |  | 2.74 (2.52-2.98)^4^ |  | 3.07 (2.82-3.34)^5^ |  | 1.70 (1.53-1.88)^6^ |
| **Patients with statin use only** |  |  |  |  |  |  |  |  |  |
| ≤5.66 | 1,411 | 237 | 29.40 (25.66-33.14) |  | 1.88 (1.61-2.18)^4^ |  | 2.00 (1.72-2.33)^5^ |  | 1.62 (1.36-1.93)^6^ |
| >5.66-9.72 | 2,580 | 309 | 16.70 (14.84-18.56) |  | 1.01 (0.88-1.16)^4^ |  | 1.06 (0.92-1.21)^5^ |  | 1.07 (0.91-1.24)^6^ |
| >9.72-15.76 | 4,896 | 585 | 15.54 (14.28-16.80) |  | 1.00 (Reference) |  | 1.00 (Reference) |  | 1.00 (Reference) |
| >15.76-24.21 | 5,247 | 735 | 18.70 (17.35-20.06) |  | 1.20 (1.08-1.34)^4^ |  | 1.20 (1.08-1.34)^5^ |  | 0.99 (0.88-1.12)^6^ |
| >24.21-35.36 | 3,179 | 534 | 26.15 (23.93-28.37) |  | 1.78 (1.58-2.00)^4^ |  | 1.78 (1.58-2.00)^5^ |  | 1.29 (1.13-1.48)^6^ |
| >35.36 | 2,362 | 571 | 49.51 (45.45-53.58) |  | 3.50 (3.11-3.93)^4^ |  | 3.47 (3.08-3.89)^5^ |  | 1.92 (1.67-2.20)^6^ |
| **Patients with fibrates use only** |  |  |  |  |  |  |  |  |  |
| ≤5.66 | 195 | 68 | 47.84 (36.47-59.21) |  | 1.37 (1.01-1.86)^4^ |  | 1.38 (1.02-1.88)^5^ |  | 1.41 (0.98-2.02)^6^ |
| >5.66-9.72 | 331 | 79 | 28.92 (22.54-35.30) |  | 0.80 (0.60-1.07)^4^ |  | 0.79 (0.59-1.05)^5^ |  | 1.07 (0.75-1.53)^6^ |
| >9.72-15.76 | 424 | 111 | 32.59 (26.53-38.66) |  | 1.00 (Reference) |  | 1.00 (Reference) |  | 1.00 (Reference) |
| >15.76-24.21 | 272 | 92 | 50.23 (39.97-60.50) |  | 1.61 (1.22-2.13)^4^ |  | 1.60 (1.21-2.12)^5^ |  | 1.31 (0.93-1.85)^6^ |
| >24.21-35.36 | 129 | 46 | 59.71 (42.46-76.97) |  | 2.27 (1.60-3.21)^4^ |  | 2.17 (1.53-3.08)^5^ |  | 1.71 (1.12-2.60)^6^ |
| >35.36 | 71 | 28 | 74.70 (47.03-102.37) |  | 2.95 (1.94-4.48)^4^ |  | 2.98 (1.96-4.54)^5^ |  | 2.07 (1.20-3.59)^6^ |
| **Cardiovascular Mortality** |  |  |  |  |  |  |  |  |  |
| ≤5.66 | 3,113 | 141 | 7.15 (5.97-8.33) |  | 1.51 (1.23-1.85)^4^ |  | 1.29 (1.05-1.59)^5^ |  | 1.40 (1.11-1.76)^6^ |
| >5.66-9.72 | 5,127 | 165 | 4.30 (3.64-4.95) |  | 0.95 (0.78-1.15)^4^ |  | 0.86 (0.71-1.05)^5^ |  | 0.93 (0.75-1.16)^6^ |
| >9.72-15.76 | 8,241 | 283 | 4.31 (3.81-4.81) |  | 1.00 (Reference) |  | 1.00 (Reference) |  | 1.00 (Reference) |
| >15.76-24.21 | 8,240 | 269 | 4.11 (3.62-4.60) |  | 0.98 (0.83-1.16)^4^ |  | 1.05 (0.88-1.24)^5^ |  | 0.93 (0.77-1.12)^6^ |
| >24.21-35.36 | 4,935 | 200 | 5.70 (4.91-6.49) |  | 1.43 (1.20-1.72)^4^ |  | 1.56 (1.30-1.88)^5^ |  | 1.13 (0.92-1.38)^6^ |
| >35.36 | 3,305 | 192 | 10.52 (9.04-12.01) |  | 2.71 (2.25-3.25)^4^ |  | 2.95 (2.45-3.55)^5^ |  | 1.64 (1.31-2.04)^6^ |
| **Patients with statin use only** |  |  |  |  |  |  |  |  |  |
| ≤5.66 | 1,411 | 51 | 6.33 (4.59-8.06) |  | 1.77 (1.28-2.44)^4^ |  | 1.86 (1.34-2.57)^5^ |  | 1.46 (1.02-2.10)^6^ |
| >5.66-9.72 | 2,580 | 60 | 3.24 (2.42-4.06) |  | 0.86 (0.63-1.16)^4^ |  | 0.88 (0.65-1.20)^5^ |  | 0.83 (0.59-1.17)^6^ |
| >9.72-15.76 | 4,896 | 135 | 3.59 (2.98-4.19) |  | 1.00 (Reference) |  | 1.00 (Reference) |  | 1.00 (Reference) |
| >15.76-24.21 | 5,247 | 157 | 4.00 (3.37-4.62) |  | 1.12 (0.89-1.41)^4^ |  | 1.12 (0.89-1.41)^5^ |  | 0.97 (0.75-1.24)^6^ |
| >24.21-35.36 | 3,179 | 115 | 5.63 (4.60-6.66) |  | 1.66 (1.29-2.13)^4^ |  | 1.69 (1.31-2.16)^5^ |  | 1.15 (0.86-1.52)^6^ |
| >35.36 | 2,362 | 123 | 10.67 (8.78-12.55) |  | 3.27 (2.55-4.18)^4^ |  | 3.30 (2.58-4.23)^5^ |  | 1.85 (1.39-2.47)^6^ |
| **Patients with fibrates use only** |  |  |  |  |  |  |  |  |  |
| ≤5.66 | 195 | 13 | 9.15 (4.17-14.12) |  | 1.12 (0.56-2.24)^4^ |  | 1.15 (0.58-2.29)^5^ |  | 1.76 (0.82-3.81)^6^ |
| >5.66-9.72 | 331 | 13 | 4.76 (2.17-7.35) |  | 0.56 (0.28-1.12)^4^ |  | 0.56 (0.28-1.12)^5^ |  | 0.62 (0.27-1.45)^6^ |
| >9.72-15.76 | 424 | 23 | 6.75 (3.99-9.51) |  | 1.00 (Reference) |  | 1.00 (Reference) |  | 1.00 (Reference) |
| >15.76-24.21 | 272 | 18 | 9.83 (5.29-14.37) |  | 1.38 (0.74-2.57)^4^ |  | 1.34 (0.72-2.51)^5^ |  | 1.13 (0.52-2.44)^6^ |
| >24.21-35.36 | 129 | 5 | 6.49 (0.80-12.18) |  | 1.22 (0.46-3.23)^4^ |  | 1.16 (0.44-3.07)^5^ |  | 0.91 (0.29-2.86)^6^ |
| >35.36 | 71 | 2 | 5.34 (-2.06-12.73) |  | 0.91 (0.21-3.86)^4^ |  | 0.92 (0.22-3.93)^5^ |  | 1.06 (0.22-4.98)^6^ |

^1^ LDL-C-SD: standard deviation of low-density cholesterol

^2^ Based on Poisson assumption, CI=confidence interval

^3^ HR= hazard ratio; CI=confidence interval

^4^ Based on Cox proportional hazard regression adjusting for general characteristics (i.e., age, and sex)

^5^ Based on Cox proportional hazard regression adjusting for the general characteristics in Model 1 plus the antidiabetic, antihypertensive, and antilipid medications presented in Table 1.

^6^ Based on Cox proportional hazard regression with all covariates included in Model 2 plus comorbidities, complications, and laboratory results presented in Table 1.

*P* values for the interaction of LDL-C-SD with statins and fibrates were 0.0708 and 0.8841, respectively.
